# Transition from Oncologist- to Therapist-Led MRI-Guided Ultra-Hypofractionated Adaptive Prostate Radiation Therapy: Evaluation of Early Clinical Outcomes

**DOI:** 10.3390/curroncol33070398

**Published:** 2026-07-03

**Authors:** Amanda Moreira, Tara Rosewall, Jennifer Dang, Aran Kim, Anna T. Santiago, Aruz Mesci, Enrique Gutierrez, Andrew Bayley, Andrew McPartlin, Rachel M. Glicksman, Alejandro Berlin, Jeff Winter, Winnie Li, Peter Chung

**Affiliations:** 1 Radiation Medicine Program, Princess Margaret Cancer Centre, University Health Network, Toronto, ON M5G 2C4, Canada; amanda.moreira@uhn.ca (A.M.); tara.rosewall@uhn.ca (T.R.); jeff.winter@uhn.ca (J.W.);; 2 Department of Radiation Oncology, University of Toronto, Toronto, ON M5G 2M9, Canada; 3 Biostatistics Core, University Health Network, Toronto, ON M5G 1X6, Canada

**Keywords:** adaptive radiotherapy, online adaptive radiotherapy, RTT roles, prostate cancer, MR-linac

## Abstract

MR-guided adaptive radiotherapy (ART) enables daily plan optimization for prostate cancer but is resource-intensive. This study evaluated dosimetric and clinical outcomes following transition from radiation oncologist (RO)-led to radiation therapist (RTT)-led MR-guided ART. All prostate cancer patients treated with MR-guided ART on a 1.5T MR-linac were retrospectively reviewed. Consecutive RO-led (September 2019–November 2021) and RTT-led (April 2022–October 2023) cohorts were compared, excluding the actual transition period. Toxicities (CTCAE v5.0), dose–volume metrics from daily adapted plans, target volume variation, and biochemical recurrence-free survival (BRFS) were analyzed. A total of 166 patients were included (78 RO-led, 88 RTT-led; median follow-up 40 and 35 months). Dosimetric differences between the cohorts were statistically small (<1%). Rates of G2+ GI adverse events were similar across all timepoints. An increase in on-treatment GU events was observed in the RTT-led cohort (G2+ 27% vs. 9%, G3 incidence *n* = 2 vs. *n* = 0), likely reflecting higher baseline urinary dysfunction; no post-treatment differences persisted. Early biochemical outcomes were comparable, with 36-month BRFS of 93.5% (RO-led) and 95.0% (RTT-led). RTT-led MR-guided ART achieved comparable dosimetric quality and early biochemical outcomes to RO-led workflows with adverse advents that resolved in the long term. With structured training and a mature practice setting, RTT-led ART represents a scalable model to support future adaptive radiotherapy practice.

## Simple Summary

MRI-guided adaptive radiotherapy (ART) allows prostate cancer treatment plans to be adjusted each day, improving precision but requiring significant staff resources. Some centers now use specially trained radiation therapists (RTTs) instead of radiation oncologists (ROs) to lead this daily adaptation. This study examined whether this change affects patient outcomes. Researchers compared prostate cancer patients treated before and after a transition from an RO-led to an RTT-led workflow. Radiation dose quality, changes in prostate size, bowel and urinary side effects, and early cancer control using PSA blood tests were reviewed. Radiation plans were of similar quality in both groups, and bowel side effects and early cancer control were comparable. Urinary side effects during treatment were more common in the RTT-led group but were linked to pre-existing urinary symptoms and did not continue after treatment. With appropriate training, RTT-led ART appears safe and supports more efficient and accessible prostate ART.

## 1. Introduction

Prostate cancer represents a substantial proportion of cancer burden in Canada [1,2]. A range of management options are available for patients with prostate cancer, including multiple radiation therapy (RT) approaches [3,4,5]. As life expectancy in Canada increases, the proportion of prostate cancer patients who may opt for RT may also increase [1,2]. More recently, increasing emphasis has been placed on patient-centered care, with quality of life and treatment-related adverse effects playing a central role in clinical decision-making.

Magnetic resonance imaging (MRI) has become increasingly important in prostate radiation therapy planning because of its superior soft-tissue contrast, enabling improved target delineation and sparing of organs at risk (OARs) [6,7]. The clinical introduction of MR-linacs (MRL) has further integrated MRI into the daily radiation therapy workflow [8]. These systems facilitate online adaptive RT (ART), a process in which images are acquired for daily contour and plan adjustments while the patient is in treatment position [9,10]. Recent evidence for ultra-hypofractionated RT in prostate cancer has established this as a standard of care and such RT regimens may be better suited for MR-guided ART [11,12,13].

Despite its potential benefits, daily online ART is resource-intensive and has historically required the presence of a full multidisciplinary team for each treatment fraction. To reduce this resource burden and increase access to care, radiation therapist (RTT)-led workflows have been implemented at various centers for MR-guided ART prostate treatments [14,15,16,17,18]. This involves task-shifting: a (re-)contouring of the target and OARs, and plan evaluation, from the radiation oncologists (ROs) to the RTTs. The existing literature on RTT-led ART workflows for prostate cancer has primarily focused on training frameworks [14,15,19], workflow feasibility, and geometric and dosimetric accuracy for RTT-driven ART [16,17,18]. However, there is a lack of data evaluating whether this task-shifting translates to comparable clinical outcomes, particularly with respect to treatment-related adverse events.

This study aimed to evaluate the impact of the transition from an RO- to RTT-led workflow on dosimetric and clinical outcomes in a prostate MR-guided ART program (institutional review board ID: QIRC 24-0883).

## 2. Materials and Methods

### 2.1. Prostate Online Adaptive Workflow

**Figure 1:**
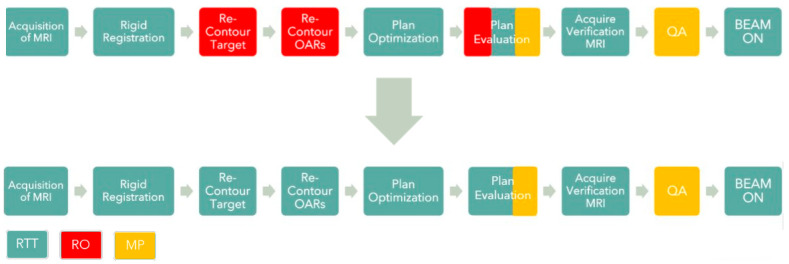
ART treatment session workflow with professional tasks assigned before (**above**) and after (**below**) task-shifting.

During each ART treatment session, the patient was positioned, MR images obtained and the target and OARs contoured; the IMRT radiation plan was then re-optimized, reviewed for quality and deliverability, and delivered based on that day’s anatomy [20]. When this workflow was first implemented, it required the presence of 3 dual certified MR-RTTs, 1 RO, and 1 Medical Physicist for each treatment session (Figure 1).

After two years of experience, during which the ROs performed key adaptive tasks, we chose to leverage the knowledge, skills and clinical judgment of the MR-RTTs to improve health system resource utilization. A structured RTT training, consolidation, evaluation and implementation process was developed for prostate ART, enabling a workflow in which ROs delegated all adaptation tasks to RTTs. This RTT-led workflow was found to be equivalent to the RO-led process in terms of accuracy and reproducibility [15]. Of note, this RTT-led workflow required an RO to be present for the first fraction, while for subsequent fractions, RO review was only required if concern was escalated from the RTTs.

### 2.2. Patient Population

All prostate ART patients treated on a 1.5T MR-linac were retrospectively reviewed. The patients were grouped into consecutive cohorts consisting of RO-led (September 2019–November 2021) and RTT-led (April 2022–October 2023). Patients treated between these 2 periods were excluded to avoid the period of transition between RO- and RTT-led workflows. Patient demographics, disease and treatment characteristics were abstracted from the medical chart.

### 2.3. Radiation Plan

All the patients were treated with 25–42.7 Gy in 5–7 alternate day fractions, using 9-field IMRT with the adapt to shape (ATS) workflow on the Unity MR-L (Elekta AB, Stockholm, Sweden). ART prescription doses of 30 Gy or less were delivered approximately 1 week after HDR brachytherapy to either the whole gland or a focal intraprostatic region. The clinical target volume (CTV) for ART included the prostate along with proximal seminal vesicle extension when clinically indicated. Planning target volume (PTV) margins were isotropic 5 mm, except for RTT-led 42.7 Gy plans (3 mm left–right, 4 mm elsewhere). All planning clinical goals for the creation of reference ART MR-L prostate plans can be found in Supplementary Materials.

### 2.4. Study Outcomes

Baseline (before brachytherapy when used) PSA, GU and GI symptoms were recorded from the clinical chart. Endpoints including PSA, acute and late toxicities (urinary and rectal CTCAE version 5.0 criteria) were retrospectively abstracted from prospective documentation entered during radiation treatment and 1, 6, 12, 24 and 36 months post-treatment. The PSA value at nadir was also identified. Biochemical recurrence-free survival (BRFS) was determined using the Phoenix criteria [21].

Target and OAR dose–volume metrics were extracted from daily re-optimized adapted treatment plans. Dose–volume metrics included 0.5 cc volume (D1 cc) for the bladder and rectum as well as dose to 50% of the volume (D50%) of the rectum and mean dose to the urethra. Dose to 95% of the planning target volume (D95) was assessed. All dosimetric values were normalized to the prescription dose to allow comparison across different dose fractionations. Additionally, the CTVs at each treatment fraction were recorded.

### 2.5. Statistical Analysis

Differences between the groups were evaluated using the Wilcoxon rank-sum test for continuous variables and chi-squared and Fisher’s exact tests for categorical variables, as appropriate. The incidence of grade (G) 2+ GU toxicities was summarized as proportions at prespecified timepoints and compared between groups using Fisher’s exact test, with Bonferroni adjustment for multiple comparisons. Univariable and multivariable logistic regression analyses were performed to identify factors associated with acute grade 2+ GU toxicity. Acute grade 2+ GU toxicity was defined as the occurrence of at least one grade 2+ genitourinary toxicity event during radiotherapy and/or at the 1-month follow-up timepoint. Covariates included cohort, age, baseline GU symptoms, prescription type, rectal spacer use, and treatment margin. Multicollinearity was assessed using variance inflation factors (VIFs). Biochemical recurrence-free survival was estimated using the Kaplan–Meier method and compared using the log-rank test, measured in months from treatment initiation to biochemical recurrence or last follow-up, whichever occurred first. A *p*-value of <0.05 was considered statistically significant.

## 3. Results

A total of 166 prostate ART patients were included, with 78 in the RO-led cohort and 88 in the RTT-led cohort. Overall, the median (min–max) follow-up in months was 40 (32–60) and 35 (18–46) for the RO-led and RTT-led cohorts, respectively. The median age was 71 years. The majority of patients (152/166, 91.6%) had Gleason 7 and T1c or T2 disease (67/166, 40.4% and 68/166, 41.0%). Table 1 summarizes the patient demographics and treatment details for each cohort. Notably, rectal spacers were used more frequently in the RO-led cohort (36% vs. 17%; *p* = 0.010). There were a higher proportion of HDR brachytherapy + ART patients in the RO cohort (47.4% vs. 17.0%; *p* < 0.001) and a higher proportion of patients receiving 42.7 Gy in the RTT cohort (60% vs. 36%; *p* < 0.001). A higher proportion of patients in the RTT cohort had baseline GU G2+ dysfunction (43% vs. 17%; *p* < 0.001).

### 3.1. Dosimetric Evaluation

**Figure 2:**
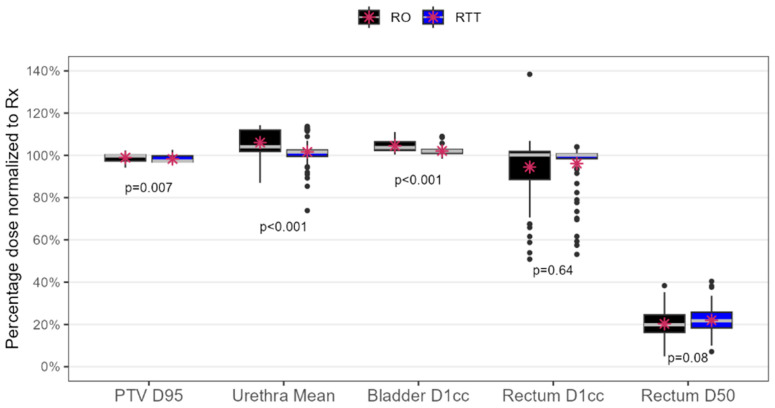
Percentage delivered doses received by PTV, bladder, urethra and rectum in the RO- and RTT-led cohorts.

Dose metrics from 1158 daily delivered plans were reviewed and summed (524 RO-led and 634 RTT-led) (Figure 2). PTV D95, bladder D1 cc and mean urethra dose were statistically significantly higher in the RO cohort (*p* < 0.007), but the absolute differences were less than 1%. For the rectum, both D1 cc (95% vs. 96%) and D50 (20% vs. 22%) were slightly higher in the RTT-led cohort, but neither reached statistical significance (*p* > 0.08).

**Figure 3:**
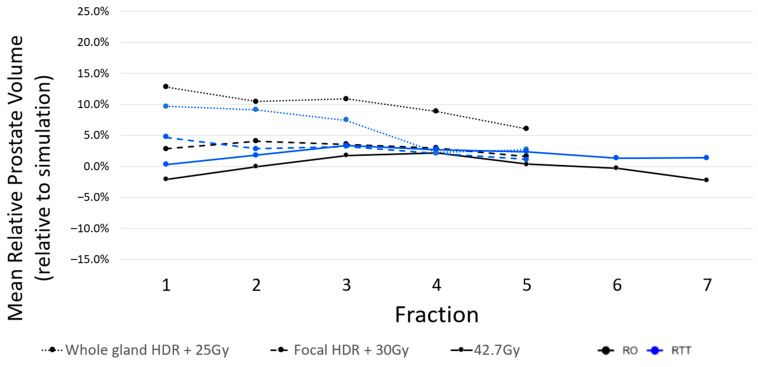
Mean percentage differences in CTV relative to simulation over the course of treatment for each prescription by cohort.

**Figure 4:**
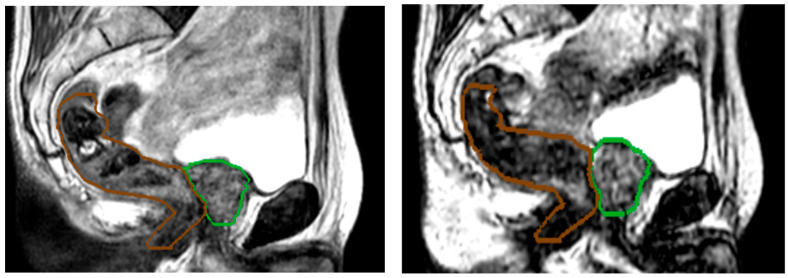
Sagittal MR illustrating a 17% decrease in CTV due to changes in rectal filling. Initial image (**left**), last fraction image (**right**). CTV in green, rectum in brown.

Median change in CTV between the initial planning images and the last fraction was 0.1% (range: −21 to 35%) in the RO cohort and 0.5% (range: −12 to 23%) in the RTT cohort. The largest increases in CTV were seen at fraction 1 and 2 for patients who received HDR brachy, in both the RO and RTT cohorts (Figure 3). Large reductions in CTV were occasionally seen in patients where rectal filling caused deformation and compression of the CTV (Figure 4).

### 3.2. Adverse Effects of RT

Overall incidence of GU and GI adverse events was low in both groups. The only instances of grade 3 adverse events were found in the RTT-led cohort (*n* = 2 GU on treatment, *n* = 1 GU at 1 month and *n* = 1 GI at 1 year). These cases were reviewed individually and no institutional dosimetric variances were exceeded during the online adapted sessions. G2+ GI toxicity was not significantly different between the cohorts during RT (0.6%), nor at 1 month (0.6%), 6 months (0.6%), 12 months (2.4%), 2 years (0.6%) or 3 years (0.6%). There was an increased incidence of G2+ GU toxicities in the RTT-led cohort only during RT (9% vs. 27%; *p* = 0.003). The G2+ GU adverse events are summarized in Table 2.

**Figure 5:**
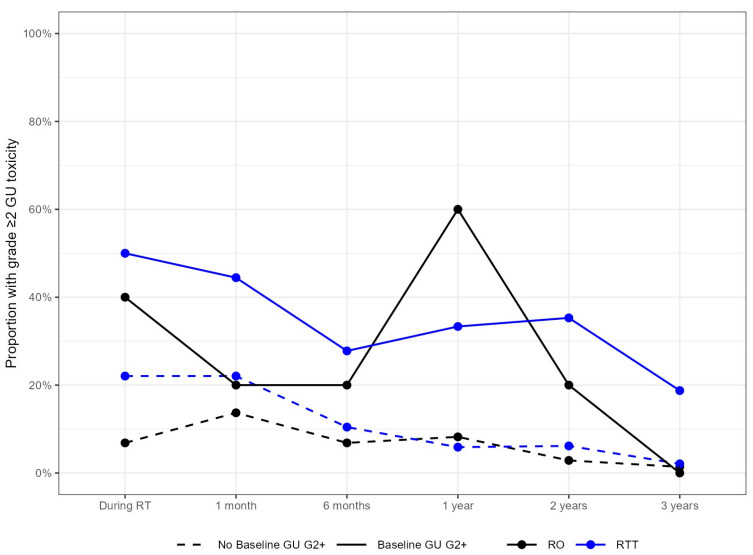
Percentage of patients reporting GU G2+ treatment-related adverse events over time by cohort and baseline urinary dysfunction.

Figure 5 summarizes GU G2+ adverse events for those individuals with and without baseline G2+ urinary dysfunction in each of the study cohorts. Univariable analysis, cohort (RO vs. RTT), baseline GU symptoms, prescription and margin reduction were found to be associated with acute G2+ GU toxicity, although upon multivariable analysis only the presence of baseline GU symptoms remained statistically significant. The results are summarized in Table 3.

### 3.3. Biochemical Outcomes

**Figure 6:**
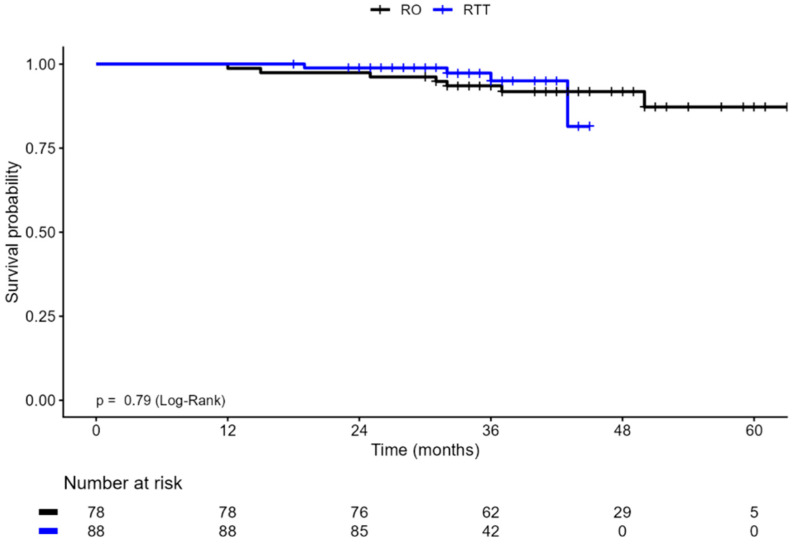
Kaplan–Meier curves for biochemical recurrence-free survival by cohort.

The mean PSA at nadir was similar between the cohorts (RO-led = 0.28 ng/mL ± 0.32 vs. RTT-led = 0.24 ng/mL ± 0.33). Overall instances of biochemical recurrence were few in both cohorts, with seven in the RO-led and four in the RTT-led group. The 36-month BRFS was 93.5% (95% CI: 88.2–99.2%) for the RO-led cohort and 95.0% (95% CI: 89.4–100.0%) in the RTT-led cohort (log rank *p* = 0.79, Figure 6).

## 4. Discussion

To date, numerous studies have considered RTT-led ART workflows for localized prostate cancer from various perspectives. The workflow itself, RTT training, and geometric and dosimetric accuracy of CTV and OAR delineation have been thoroughly investigated and confirm the technical accuracy of RTT-led adaptation with MRL [14,15,16,17,18]. Recently an economic lens has been added that confirms the systems-level benefit of task-shifting from ROs to RTTs [22]. To our knowledge, this manuscript describes the first evaluation of the impact of an RTT-led workflow on clinical outcomes following MR-guided ART beyond summaries of acute adverse events. We believe this early evaluation is timely given the substantial increase in international utilization of RTT-led workflows for ART.

A total of 166 prostate ART patients were included in this analysis, with 78 in the RO-led cohort and in the 88 RTT-led cohort and a median follow-up of 40 months (32–60) and 35 months (18–46), respectively. Overall, the percentage of patients reporting G2+ adverse events herein compares favorably with the literature describing adverse events following similar ultra-hypofractionated SBRT regimens [13,23,24] and with other prostate MR-guided ART studies, regardless of specific discipline-led workflow [25,26], particularly when 51% of our cohort also received pre-SBRT HDR brachytherapy.

The overall incidence of G2+ GI adverse events was not significantly different between the RTT- or RO-led cohorts, across all timepoints. However, we did find an increase in G2+ GU adverse events during the treatment course in the RTT-led cohort. Errors in RTT CTV and/or bladder delineation (particularly at the bladder/prostate interface) could result in an increase in GU toxicity. But this research confirms the similarity between RTT and RO contours and consistently delivered dosimetry. It is likely that differences in the patient or disease characteristics of the non-randomized cohorts may have resulted in this increase. For example, 43% of patients in the RTT-led cohort had baseline G2+ urinary symptoms, but only 17% in the RO-led cohort. This is supported by the results of our multivariable analysis, on which this was the only covariate that remained statistically significantly associated with GU 2+ toxicity. Notably, cohort (RO-led vs. RTT-led) retained the marginal trend towards statistical significance (*p* = 0.059) which could be due to over 40% of the RTT-led patients also experiencing G2+ GU baseline symptoms. A future study with larger sample sizes would help definitively decouple these two variables. The impact of baseline urinary function on G2+ GU adverse events is clearly seen in our study, and is also found in the literature [24,27,28,29]. After the ART course was completed, there were no differences in G2+ GU adverse events between the cohorts. Of note, the only instances of grade 3 adverse events were found in the RTT-led cohort (*n* = 2 GU on treatment, *n* = 1 GU at 1 month and *n* = 1 GI at 1 year). This trend warrants careful observation as median follow-up length increases.

The mean PSA at nadir was similar between the cohorts and the instances of biochemical recurrence were few. The 36-month BRFS was also similar between the cohorts (93.5% and 95.0%). Although it is early to report BRFS, these findings are promising when compared to studies that have reported BRFS at similar timepoints for similar dose regimens [30,31,32]. These results should also be viewed considering the non-randomized use of ADT, which while not statistically significantly different was ~12% higher in the RTT cohort. While longer follow-up is needed to confirm sustained biochemical control, these early findings indicate that an RTT-led adaptive workflow did not compromise early biochemical outcomes.

The dose–volume metrics for the PTV, urethra, bladder and rectum for 1158 ART plans demonstrated minimal difference between RTTs and ROs within a mature RTT-led ART program. These findings are similar to those previously reported by cancer centers describing their early experiences with clinical implementation of RTT-led ART [15,16,17], and demonstrates the long-term stability of RTT delineation skills following the successful completion of a three-phase training strategy [15].

On average, CTVs varied by less than 5% across the treatment course for those receiving 42.7 Gy ART and focal HDR with 30 Gy ART in both RTT- and RO-led cohorts. This is substantially less variation than reported by others [33] and may be related to our inclusion of patients who had received ADT. Only those who received whole gland HDR with 25 Gy ART demonstrated systematic increases in CTV compared to the initial planning images, that reduced slowly over time. This suggests that mechanical trauma and local inflammation associated with whole gland HDR may have caused initial prostate edema which resolved over the 1.5-week ART treatment course. Importantly, the lack of variation between professional groups provides reassurance that the RTTs did not introduce systematic target volume inflation or geographic miss which could have led to the observed short-term increase in GU events.

The findings of this study should be interpreted in context with the strengths and limitations of its design. This was a non-randomized investigation of consecutive cohorts of patients. Thus, difference in time periods could also reflect a difference in program maturity with a bias towards the RTT cohort. However, this bias may be minimal as both professional groups were new to MR-guided online ART at the start of their two-year window. The two cohorts (purposefully differentiated by RTT or RO adaptors) may also contain unintentional differences in variables that could influence the primary outcomes. We have endeavored to document those differences where possible but acknowledge that other confounders may still be present such as dose heterogeneity between cohorts, their resulting different BEDs and treatment-related adverse event profiles. A strength of this design was the use of the transition period between the cohorts that enabled the exclusion of patients treated during a period of training for task-shifting. Furthermore, we were able to include all patients treated during the study time periods, representing real world clinical practice at our institution. It should be noted that both the RTTs and ROs were practicing within a mature MR-L ART program with established procedures and workflow strategies. Also, the RTTs were all dual certified in RT and MRI, working in a jurisdiction that includes dosimetry in entry to practice education, and had all successfully completed an additional three-phase MR-L adaptive training program [15]. Thus, the RTT-led outcomes described herein may not be generalizable to those without formal training, or those practicing in a new MRL-ART program. Furthermore, patient selection is paramount to safe implementation. In this RTT-led workflow the RO was present for the first fraction and only required for subsequent fractions if the patient was deemed high-risk (e.g., severe intestinal peristalsis), although this did not occur in this cohort. Despite these limitations, our data provides an important first indication that RTT-led MR-guided adaptive radiotherapy can be delivered without a negative impact on short-term clinical outcomes. Although long-term follow-up and supporting evidence from other institutions remains essential, this data provides a measure of confidence that clinical outcomes are stable when shifting adaptive responsibilities toward RTTs. The success of that shift in responsibility will ultimately determine the efficiency and accessibility of ART [34,35,36,37,38].

## 5. Conclusions

In this non-randomized comparative evaluation of MR-guided adaptive radiotherapy for localized prostate cancer, the RTT-led workflow demonstrated comparable dosimetric quality and clinical outcomes to the RO-led approach with GU adverse advents that initially increased but resolved in the long term. Dual certified RTT-led MR-guided ART has been integrated into a mature and highly standardized clinical ART program and may facilitate a scalable pathway to improve treatment efficiency, optimize clinician workload, and enhance access to ART.

Ongoing follow-up with larger sample sizes and multi-institutional validation will be essential to confirm long-term safety and oncologic outcomes and to inform the broader implementation of adaptive task-shifting strategies.

## Figures and Tables

**Table 1:** Distribution of demographic and treatment variables from prostate ART patients divided by workflow type with results of Wilcoxon rank-sum (continuous) or chi-square tests (categorical).

	Full Sample (*n* = 166)	RO Cohort (*n* = 78)	RTT Cohort (*n* = 88)	*p*-Value
Age				0.46
Median (Min, Max)	71 (49, 89)	72 (54, 88)	71 (49, 89)	
Prostate Volume				0.59
Median (Min, Max)	41.9 (16.3, 120.1)	42.7 (20.7, 120.1)	40.2 (16.3, 106.2)	
Baseline PSA				0.91
Median (Min, Max)	8.50 (0.1, 123.0)	8.88 (0.1, 27.23)	8.38 (0.4, 123.0)	
Gleason Score				0.051
6 (%)	6 (3.7)	5 (6.4)	1 (1.2)	
3 + 4 (%)	115 (70.6)	48 (61.5)	67 (78.8)	
4 + 3 (%)	37 (22.7)	23 (29.5)	14 (16.5)	
8 (%)	2 (1.2)	1 (1.3)	1 (1.2)	
9 (%)	3 (1.8)	1 (1.3)	2 (2.4)	
Missing	3	0	3	
Rectal Spacer				**0.010**
No spacer (%)	123 (74.1)	50 (64.1)	73 (83.0)	
Spacer (%)	43 (25.9)	28 (35.9)	15 (17.0)	
Hormone Therapy				0.14
No (%)	91 (54.8)	48 (61.5)	43 (48.9)	
Yes (%)	75 (45.2)	30 (38.5)	45 (51.1)	
Baseline Genitourinary Symptoms (G2+)				**<0.001**
No (%)	115 (69.3)	65 (83.3)	50 (56.8)	
Yes (%)	51 (30.7%)	13 (16.7)	38 (43.2)	
RX				**<0.001**
Whole Gland HDR + 25.0 Gy (%)	33 (19.9)	13 (16.7)	20 (22.7)	
Focal HDR + 30.0 Gy (%)	52 (31.3)	37 (47.4)	15 (17.0)	
42.7 Gy (%)	81 (48.8)	28 (35.9)	53 (60.2)	

Bolded *p*-Values found to be statistically significant.

**Table 2:** Incidence of patients reporting GU G2+ toxicities at each timepoint by cohort, presented as n/N (%) with denominators reflecting patients with available data. *p*-values were calculated using Fisher’s exact test with Bonferroni adjustment for multiple comparisons.

Timepoint	RO Cohort *n* = 78	RTT Cohort *n* = 88	*p*-Value	Adjusted *p*-Value (Bonferroni)
During RT	7/78 (9%)	24/88 (27%)	0.003	0.016
1 month	11/78 (14%)	24/88 (27%)	0.06	0.33
6 months	6/78 (8%)	12/87 (14%)	0.32	>0.99
1 year	9/78 (12%)	10/88 (11%)	>0.99	>0.99
2 years	3/75 (4%)	10/84 (12%)	0.09	0.52
3 years	1/77 (1%)	4/65 (6%)	0.18	>0.99

**Table 3:** Factors associated with acute G2+ GU toxicity: univariable and multivariable logistic regression analyses.

Covariate	Univariable		Multivariable	
	OR (95% CI)	*p*	Adjusted OR (95% CI)	*p*
Cohort				
RO-led	Reference		Reference	
RTT-led	2.99 (1.44, 6.57)	0.004	3.60 (0.98, 14.63)	0.059
Age	1.01 (0.96, 1.07)	0.60	0.99 (0.94, 1.05)	0.81
Baseline GU symptoms				
No symptoms	Reference		Reference	
Symptoms	5.18 (2.49, 11.08)	<0.001	3.95 (1.48, 11.24)	0.007
Prescription type				
4270 (SBRT alone)	Reference		Reference	
2500 + Brachy	0.23 (0.06, 0.67)	0.013	0.28 (0.04, 1.63)	0.17
3000 + Brachy	0.36 (0.15, 0.81)	0.017	0.68 (0.16, 2.79)	0.59
Rectal spacer				
No spacer	Reference		Reference	
Spacer	0.69 (0.29, 1.55)	0.39	0.91 (0.35, 2.31)	0.85
Margin				
5 mm	Reference		Reference	
Reduced (3/4 mm)	3.56 (1.73, 7.45)	<0.001	0.44 (0.08, 2.42)	0.35

Acute grade ≥ 2 GU toxicity was defined as the occurrence of at least one grade ≥ 2 genitourinary toxicity event during radiotherapy and/or at the 1-month follow-up timepoint (43/166 patients, 25.9%). No evidence of substantial multicollinearity was observed (all VIFs < 2.3).

## Data Availability

The raw data supporting the conclusions of this article will be made available by the authors on request.

## Supplementary Materials

The following supporting information can be downloaded at https://www.mdpi.com/article/10.3390/curroncol33070398/s1, Table S1: Clinical guidelines for prostate ART reference plan creation.

## Abbreviations

The following abbreviations are used in this manuscript:

ART	Adaptive radiation therapy
BRFS	Biochemical recurrence-free survival
GI	Gastrointestinal
GU	Genitourinary
MRI	Magnetic resonance imaging
MRL	MR-linac
RTT	Radiation therapy
RO	Radiation oncologist
