# Supplementary material for: Transition from Oncologist- to Therapist-Led MRI-Guided Ultra-Hypofractionated Adaptive Prostate Radiation Therapy: Evaluation of Early Clinical Outcomes
Source: Curr Oncol. 2026 Jul 3;33(7):398. doi: 10.3390/curroncol33070398 (PMC13408532; doi:10.3390/curroncol33070398)
Supplement: Supplementary file 1 [file curroncol-33-00398-s001.zip › curroncol-4336653-supplementary.pdf]

Supplemental Material

Supplemental S1. Clinical Guidelines for prostate ART reference plan creation

**Structure**	**Metric**	**Criteria**		
		**2500cGy**	**3000cGy**	**4270cGy**
**CTVp**	D99	> 2500 cGy	-	> 4270 cGy
**CTVp**	D95	-	> 3300 cGy	-
**PTVp**	D2	< 2700 cGy	< 3500 cGy	< 4610 cGy
**PTVp**	D95	> 2500 cGy	> 3000 cGy	-
**PTVp**	D98	> 2375 cGy	> 2850 cGy	> 4060 cGy *
**Rectum**	D50	< 835 cGy	< 1000 cGy	< 1000 cGy
**Rectum**	D20	< 1665 cGy	< 2000 cGy	< 2400 cGy
**Rectum**	D1cc	< 2500 cGy	< 3000 cGy	< 4270 cGy
**Bladder**	D40	< 1250 cGy	< 1500 cGy	< 1800 cGy
**Bladder**	D5cc	< 2500 cGy	< 3000 cGy	< 4270 cGy
**Femur_L**	D5	< 1000 cGy	< 1200 cGy	< 1800 cGy
**Femur_R**	D5	< 1000 cGy	< 1200 cGy	< 1800 cGy
**SmallBowel**	D1cc	< 2085 cGy	< 2500 cGy	< 3000 cGy
**SmallBowel**	D5cc	< 1250 cGy	< 1500 cGy	-
**LargeBowel**	D1cc	< 2085 cGy	< 2500 cGy	< 3000 cGy
**LargeBowel**	D5cc	< 1250 cGy	< 1500 cGy	-
**PenileBulb**	D50	< 2000 cGy	< 2400 cGy	< 3420 cGy
**PenileBulb**	D1cc	< 2500 cGy	< 3000 cGy	< 4270 cGy
**Urethra**	D50	< 2700 cGy	< 3500 cGy	< 4500 cGy

*D99
